# Investigating cytokinesis failure as a strategy in cancer therapy

**DOI:** 10.18632/oncotarget.13556

**Published:** 2016-11-24

**Authors:** Callum McKenzie, Pier Paolo D’Avino

**Affiliations:** ^1^ Department of Pathology, University of Cambridge, Cambridge, CB2 1QP, UK

**Keywords:** citron kinase, cytokinesis failure, chromosomal instability, polyploidy

## Abstract

Effective therapeutics exploit common characteristics shared amongst cancers. As many cancers present chromosomal instability (CIN), one possible approach to treat these cancers could be to increase their CIN above a threshold that would affect their viability. Here, we investigated whether causing polyploidy by cytokinesis failure could represent a useful approach. We show that cytokinesis failure caused by depletion of Citron kinase (CIT-K) dramatically decreased cell proliferation in breast, cervical and colorectal cancer cells. CIT-K depletion activated the Hippo tumor suppressor pathway in normal, but not in cancer cells, indicating that cancer cells have evolved mechanisms to bypass this control. CIT-K depleted cancer cells died via apoptosis in a caspase 7 dependent manner and, consistent with this, p53-deficient HCT116 colon carcinoma cells failed to induce apoptosis after cytokinesis failure. However, other p53-mutated cancer cells were able to initiate apoptosis, indicating that cytokinesis failure can trigger apoptosis through a p53-independent mechanism. Finally, we found that actively dividing and, in some cases, polyploid cancer cells were more susceptible to CIT-K depletion. In sum, our findings indicate that inducing cytokinesis failure could be a promising anti-cancer therapeutic approach for a wide range of cancers, especially those characterized by fast cell proliferation and polyploidy.

## INTRODUCTION

Chromosomal instability (CIN) is a hallmark of many cancers [1]. CIN is characterized by recurrent chromosomal changes that contribute to tumorigenesis by altering the balance of critical growth and death pathways. Although its role in cancer onset is still debated, CIN has been implicated in cancer evolution, diversification and heterogeneity, is associated with poor clinical outcome and drug resistance, and has been suggested to play a role in the development of metastases [2–4]. CIN can be classified either as structural (alterations in chromosome structure, such as translocations, inversions, duplications, etc.) or numerical (alterations in chromosome numbers, i.e. aneuploidy and polyploidy), but they frequently occur together in many cancers. Many studies have focused on understanding the mechanisms that generate aneuploidy in order to shed light into the genesis of numerical CIN [5, 6]. It is becoming increasingly evident that polyploidy also plays an important role in promoting tumorigenesis, CIN and drug resistance, thus contributing to cancer evolution and clonal heterogeneity [7–9]. There have been suggestions that targeting CIN might be a useful approach for cancer treatment [10, 11] and one possible strategy could be to increase polyploidy by interfering with cell division in cancer cells above a threshold that would be incompatible with cell viability. Such a strategy would specifically target highly proliferating CIN cancer cells and thus be potentially less toxic for normal tissues.

With this hypothesis in mind, we have investigated whether causing polyploidy via cytokinesis failure could be a valid strategy in promoting cell death in cancer cells. To this aim, we decided to provoke cytokinesis failure by targeting the serine/threonine kinase citron kinase (CIT-K) for the following reasons. First, CIT-K is specifically required only during the late stages of cytokinesis for the organization and function of the midbody, an organelle required for the final separation, or abscission, of the two daughter cells [12–16]. This specific late mitotic function makes CIT-K an interesting target in the light of recent studies indicating that targeting post-metaphase events is a more effective cancer treatment than other anti-mitotic agents [17, 18]. Second, CIT-K depletion by RNAi has already been shown to decrease proliferation of prostate and hepatocellular cancer cells and to inhibit tumor growth in xenograft mice models [19, 20]. Third, genetic analysis in *Drosophila* indicated that proliferating tissues from mutants carrying strong allelic combinations of the CIT-K orthologue were highly polyploid (8N or more), misshapen, and smaller than their wild type counterparts. By contrast, the tissues of animals carrying weaker allelic combinations were tetraploid and normal in shape and size [21]. These results indicate that, at least in *Drosophila*, low level of cytokinesis failure/polyploidy caused by hypomorphic CIT-K allelic combinations did not affect organ growth and structure, whereas multiple cytokinesis failures led to cell death and impaired tissue development. Therefore, it is conceivable that CIT-K inhibition could selectively affect the proliferation of very actively dividing cells. Finally, CIT-K could be potentially targeted by small molecule inhibitors designed to interfere with its ATP binding pocket, an approach successfully used to generate inhibitors of other cell division kinases of the Aurora and Polo-like kinase families that are currently in clinical trials for the treatment of various cancer pathologies [22].

We analyzed the effects of depleting CIT-K through RNA interference (RNAi) in a panel of cancer cell lines originating from breast, colorectal and cervical tissues. We found that, in all cell lines, CIT-K depletion caused cytokinesis failure, which decreased cell proliferation and triggered apoptosis via activation of executioner caspase 7. We also investigated why certain cancer cell lines elicited different sensitivities to CIT-K depletion and found that actively dividing cells and, to some extent, polyploid cells were significantly more sensitive to cytokinesis failure. These findings position CIT-K as a promising anti-proliferative target for the potential treatment of a wide range of cancers characterized by active cell proliferation and polyploidy.

## RESULTS

### CIT-K is over-expressed and mutated in a variety of cancers, but does not possess oncogenic activity

**Figure 1:**
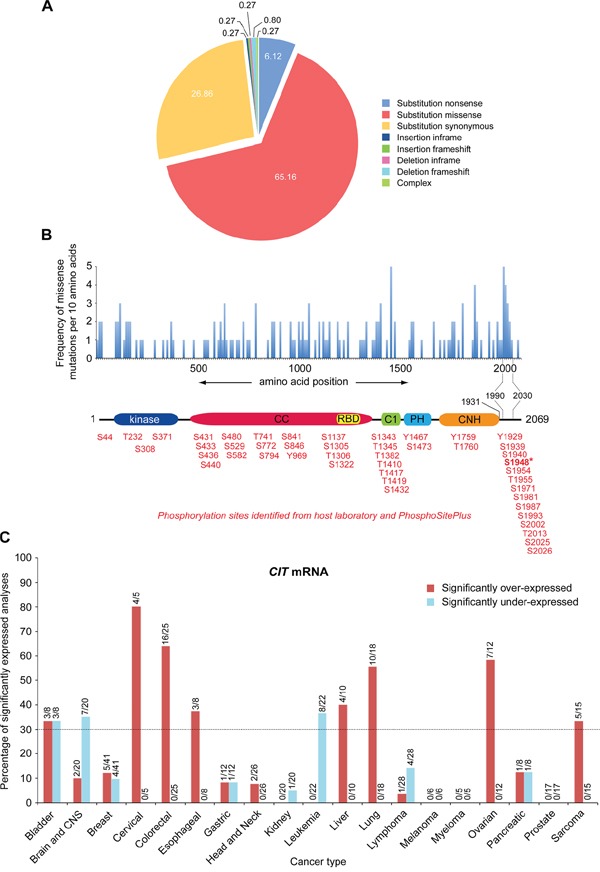
Cancers display even distribution of somatic mutations across the *CIT* gene and predominately over-express *CIT* mRNA **A**. *CIT* point mutations identified from the COSMIC database (gene ID: CIT_ENST00000261833) were categorized into types of mutations. The percentage each mutation type contributes is noted within each segment. **B**. *CIT* substitution missense mutations across the gene were calculated and mapped per 10 amino acids. A schematic representation of CIT-K is shown below the mutation histogram to visualise the location of the mutations. Phosphorylation sites identified from our laboratory and PhosphoSitePlus are listed below the CIT-K protein structure. S1948 is in bold and indicated by an asterisk to highlight that it was found mutated in the COSMIC database. CC, coiled coil; RBD, Rho/Rac binding domain; C1, cysteine-rich motif; PH, pleckstrin homology; CNH, Citron-NIK homology. **C**. *CIT* mRNA from cancerous tissue was datamined from the Oncomine™ database and compared against corresponding normal tissues to observe any over- or under-expression. All of the datasets on Oncomine™ were included in the analysis, whether they showed a significant (*p* < 0.001) difference in *CIT* mRNA expression or not. The bars show how many datasets showed either significant over- or under-expression, with the numbers above each bar indicating exactly how many of those datasets demonstrated a significant difference.

We first sought to establish whether CIT-K had any oncogenic properties to eliminate the possibility of complications in our study linked to a potential ‘oncogene addiction’ effect. To this goal, we first analyzed the type and frequency of *CIT* mutations found in cancers from the catalogue of somatic mutations in cancer (COSMIC) database [23]. *CIT* was mutated in a low percentage (<5%) of cancers spread across a range of tissues (Supplementary Figure S1). Of these point mutations, the majority (65.16%) were missense mutations, just over a quarter (26.86%) were synonymous, and 6.12% were nonsense mutations (Figure 1A). The remainder of mutations included either insertions or deletions, however these were at a very low frequency. Mapping the missense mutations on the CIT-K protein sequence revealed that there was an even distribution of mutations across the gene, with no single hotspot (Figure 1B). However, there was an accumulation of mutations in the C-terminus of CIT-K between amino acids 1990 and 2030. Interestingly, the C-terminal tail downstream of the CNH domain is subject to heavy phosphorylation, as indicated by our previous results [13] and by the data available at the PhosphoSitePlus database [24]. Although only one of these phosphosites was found mutated in the COSMIC database (S1948I, highlighted in bold in Figure 1B), this evidence could nonetheless suggest that the C-terminal tail may have an important role in the regulation and/or function of CIT-K and could explain why it is often mutated in cancers.

We next wanted to develop a better understanding of how *CIT* mRNA expression varies in different cancers. To address this, we collected data from Oncomine™, a large database storing publically available cancer gene expression datasets [25]. In order to get the best representation of *CIT* mRNA expression in cancers, we collated data from all of the datasets available that compared cancer tissue versus the corresponding normal tissue and identified the datasets reporting significantly (*p* < 0.001) over- or under-expressed *CIT* mRNA. We set an arbitrary threshold value of 30%, above which we considered there was a meaningful amount of datasets showing significant *CIT* mRNA over- or under-expression for that specific cancer type. This meta-analysis revealed that *CIT* mRNA was significantly over-expressed in bladder, cervical, colorectal, esophageal, liver, lung, ovarian and sarcoma cancers (Figure 1C). Conversely, *CIT* mRNA was significantly under-expressed in bladder, brain/CNS, and leukemia cancers (Figure 1C).

**Figure 2:**
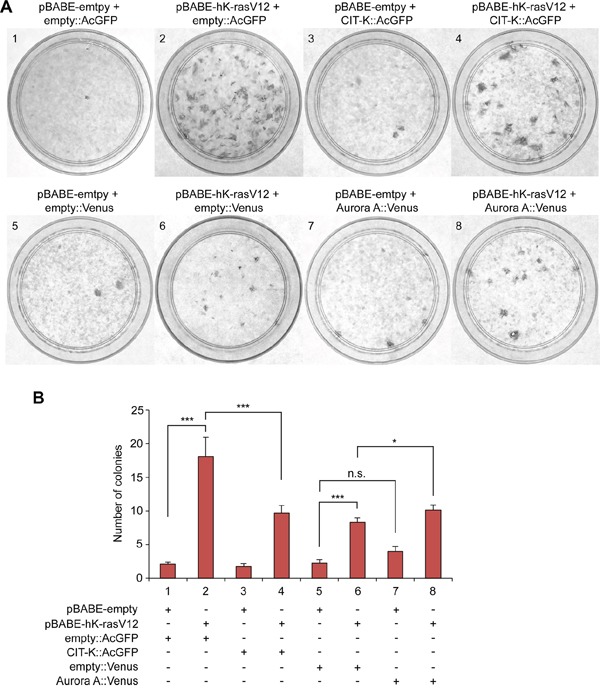
Over-expression of CIT-K does not increase colony formation **A**. NIH3T3 cells were transfected with AcGFP empty vector, CIT-K::AcGFP, Venus empty vector and Aurora A::Venus in combination with either pBABE empty vector or pBABE-hK-rasV12. Cells were cultured for 2 weeks, fixed, stained with crystal violet and colonies counted. Images displayed show black and white contrast of crystal violet stain. The numbers on the top left of each condition denote the transfection treatment as shown in B. **B**. Quantification of the colonies counted from A. For each condition n=4, the experiment was completed in triplicate (pooled data, n=12). *p < 0.05, ***p < 0.001 (Student's *t*-test), n.s., not significant, error bars represent standard error of the mean (SEM).

To understand whether *CIT* over-expression could potentially translate into tumorigenic behaviour, we assessed whether CIT-K had any oncogenic properties. To this aim, we tested whether over-expression of CIT-K could promote proliferation in a colony formation assay in murine fibroblasts NIH3T3 cells. NIH3T3 cells lost contact inhibition and developed colonies when transfected with the constitutive active mutant form of human K-rasV12 (hK-rasV12) (Figure 2A, 2B – condition 2). By contrast, over-expression of CIT-K in the absence of hK-rasV12 did not increase colony formation compared to control conditions (Figure 2A, 2B – condition 3) and combined over-expression of CIT-K and hK-rasV12 significantly decreased colony formation (Figure 2A, 2B – condition 4). Over-expression of the mitotic kinase Aurora A – known to have oncogenic activity and thus used as control [26, 27] – slightly increased colony formation (Figure 2A, 2B – condition 7) and a significant increase in colony formation was observed following over-expression of Aurora A::Venus with hK-rasV12 (Figure 2A, 2B – condition 8).

In conclusion, the combined analyses of *CIT* mutations and mRNA expression in cancers (Figure 1), and the effects of its over-expression data in NIH3T3 cells (Figure 2), do not support the possibility of an oncogenic role for CIT-K.

### CIT-K depletion causes cytokinesis failure in multiple cell lines and activates the Hippo tumor-suppressor pathway in normal, but not cancer cells

It has been reported that CIT-K knockout mice show polyploidy and cytokinesis failure only in the central nervous system and in testes [28, 29]. Thus, to understand the requirement of CIT-K for cytokinesis in human cells of different origins, we depleted CIT-K in various cancerous and non-cancerous immortalized cell lines, characterized by diverse karyotypes and p53 status (Table 1) [30–33].

**Table 1:** Detailed characterisation of cell lines analyzed

Cell line	p53	Ploidy	CIN	Multi-nucleation fold diff.	Proliferation fold diff.	Apoptotic fold diff.	Doubling time (hours)	CIT-K expression	TUBB3 expression
hTERT-RPE1	WT	46	-	5.14	1.543	2.166	29	0.711	29.51
HB4a	WT	69	n/a	9.90	1.967	1.683	27.7	0.299	6.711
CAL51	WT	46	n/a	8.85	1.481	1.305	28.2	0.647	0.736
MDA-MB-231	R280K	64	n/a	3.40	1.481	1.575	31.8	1.943	0.471
HeLa Kyoto	HPV^+ve^	67	+	7.10	2.248	2.271	25.9	0.862	0.769
ME180	HPV^+ve^	63	n/a	17.79	2.243	1.855	22.2	1.624	0.075
HCT116	WT	45	-	3.56	1.196	1.162	24.7	0.121	1.488
LoVo	WT	49	-	n/a	1.842	n/a	n/a	0.454	0.233
LS147T	WT	45	-	n/a	2.197	n/a	n/a	0.028	0.047
VACO5	R282W	46	-	21.83	2.102	2.193	17.7	0.111	0.104
Caco2	E204X	96	+	n/a	1.374	n/a	n/a	0.557	1.258
HT29	R273H	71	+	n/a	1.435	n/a	n/a	0.705	0.391
SW620	R273H	50	+	n/a	1.226	n/a	n/a	0.485	1.545
SW403	E51X	68	+	n/a	1.676	n/a	n/a	7.697	2.243
HCT116 p53^+/+^	WT	n/a	-	8.77	1.443	n/a	32.9	n/a	n/a
HCT116 p53^-/-^	Null	n/a	n/a	2.65	1.561	n/a	25.9	n/a	n/a
HCT116 2N	WT	2N (46)	-	n/a	1.570	n/a	n/a	n/a	n/a
HCT116 4N	WT	4N (92)	n/a	n/a	1.725	n/a	n/a	n/a	n/a

Nine parameters were assessed: p53 status; ploidy; CIN; multinucleation, proliferation and apoptosis fold difference (fold diff.) following CIT-K depletion; doubling time; CIT-K and TUBB3 protein expression levels. The ploidy of each cell line was obtained from the American Type Culture Collection or the references stated in the text. CIT-K and TUBB3 expressions were normalized to tubulin. WT, wild type; n/a, not available.

**Figure 3:**
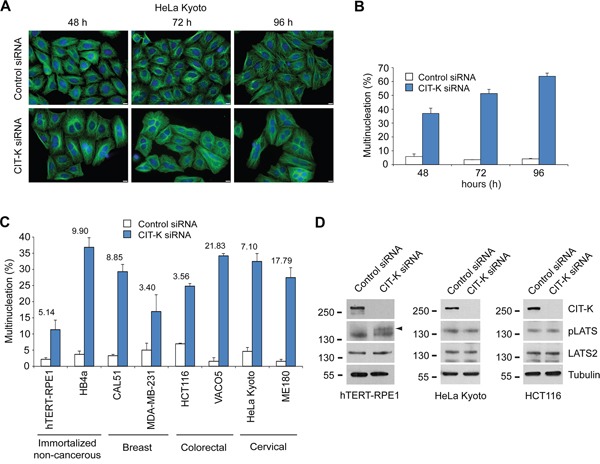
CIT-K is essential for the completion of cytokinesis in a panel of cancer cell lines **A**. HeLa Kyoto cells were plated on coverslips and treated with a siRNA against either a random sequence (control) or CIT-K for 48, 72 and 96 hours (h), where cells were fixed and stained to detect tubulin (green) and DNA (blue). Bars, 10 μm. **B**. Quantification of multinucleation increase from the experiment shown in A. At least 600 cells were counted for analysis in each experiment, n=3, error bars represent SEM. **C**. Quantification of the percentage of multinucleated cells (of cell lines listed) that were treated with either control or CIT-K siRNA for 48 h. Cells were fixed and stained to detect tubulin (green), phalloidin (red) or DNA (blue). The fold difference between control and CIT-K siRNA-treated cells is listed above each cell line. At least 600 cells were counted for analysis in each experiment, n=3, error bars represent SEM. **D**. hTERT-RPE1 (left panel), HeLa Kyoto (middle panel) and HCT116 (right panel) cells were treated with either control or CIT-K siRNA for 48 h, harvested and protein extracts analyzed by Western blot to detect CIT-K, phosphorylated LATS (pLATS), LATS2 and tubulin. The numbers on the left indicate the sizes in kilodaltons of the molecular mass marker.

We first analyzed the effect of CIT-K depletion by RNAi in HeLa Kyoto cervical cancer cells over a 96-hour time course (Figure 3A). Depleting CIT-K for 48 hours caused ∼37% of cells to become multinucleated and 72 and 96 hour treatments increased multinucleation to ∼51% and ∼64%, respectively (Figure 3B). We then investigated whether CIT-K was essential for cytokinesis in a panel of cell lines comprising both non-cancerous immortalized cell lines (hTERT-RPE1 and HB4a) and cancer cell lines from breast (CAL51 and MDA-MB-231), colorectal (HCT116 and VACO5) and cervical (HeLa Kyoto and ME180) origins (Table 1). Western blot analysis confirmed an almost complete depletion of CIT-K in all cell lines after 48 hours, and persisting up to 96 hours, after siRNA treatment (Supplementary Figure S2). In all cell lines tested, CIT-K depletion caused significant cytokinesis failure after only 48 hours of treatment, with VACO5 and ME180 cells experiencing the largest increases of multinucleation (Figure 3C, Supplementary Figure S3). We also confirmed that CIT-K siRNA depletion activated the Hippo tumor suppressor pathway through phosphorylation of LATS2 in hTERT-RPE1 cells (Figure 3D, left panel), as previously demonstrated in other cases of cytokinesis failure [34]. However, the Hippo tumour suppressor pathway after CIT-K depletion was not activated in HeLa Kyoto or HCT116 cancer cell lines (Figure 3D, middle and right panels), suggesting that at least some cancer cells have adapted to inhibit or bypass this control mechanism.

In conclusion, our results indicate that CIT-K depletion triggers cytokinesis failure in many cell types, independently of their origin and other characteristics such as ploidy and p53 status.

### CIT-K RNAi inhibits cell proliferation and induces cell death through apoptosis

**Figure 4:**
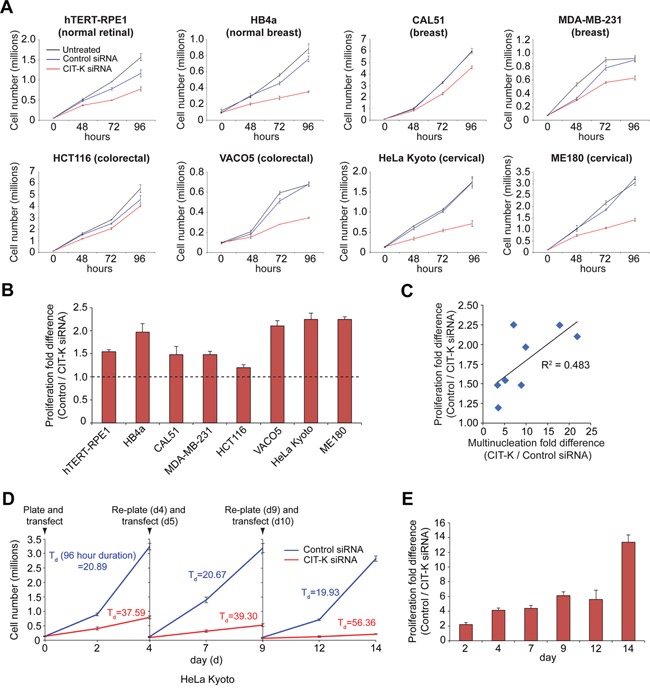
Depleting CIT-K decreases cell proliferation in cancer cells **A**. Cell lines were plated the day prior to transfection and were either untreated or treated with control or CIT-K siRNA at day 0 for up to 96 hours. Cell number was calculated at each time point and an aliquot of cells taken for Western blot analysis. Each condition was performed in triplicate, error bars represent SEM. **B**. Quantification of fold difference between control and CIT-K-siRNA treated cells at 96 h, n=3 (pooled data from A, n=9), error bars represent SEM. The dotted line represents a fold difference of 1 (being no difference in proliferation compared to control). Values above this line indicate a decrease in proliferation. **C**. Correlation analysis of multinucleation and proliferation fold differences. The values used for analysis can be found in Table 1. The Pearson correlation coefficient (R^2^) is listed next to the linear trend line. **D**. HeLa Kyoto cells were treated with either control or CIT-K siRNA for up to 96 h, where cells were counted, re-plated and transfected the subsequent day, for up to 14 days. At each time point an aliquot of cells was taken for Western blot analysis. The doubling time of cells (T_d_) was calculated over a 96 hour time period (day 0 to 4, day 5 to 9 and day 10 to 14) and listed next to the cell proliferation curve. Each condition was performed in triplicate, error bars represent SEM. **E**. Quantification of the experiment shown in D showing the proliferation fold difference between control and CIT-K siRNA-treated cells at each time point.

As our findings indicated that multinucleation increased following CIT-K depletion, we investigated how cytokinesis failure affected cell proliferation. We found that cell proliferation decreased in all cell lines after CIT-K RNAi when compared against control cells treated with a scrambled siRNA (Figure 4A, Supplementary Figure S2). We calculated the fold difference in proliferation after 96 hours of CIT-K siRNA treatment (Figure 4B, Table 1), which revealed that the colorectal cell line VACO5 and the cervical cell lines HeLa Kyoto and ME180 were most affected (fold difference greater than 2). We also found a positive correlation (R^2^ = 0.483) between the increase in multinucleation and the decrease in proliferation in CIT-K depleted cells (Figure 4C).

To understand the effects of long term CIT-K depletion, we performed several rounds of siRNA transfection and measured cell number at various time points over two weeks (Figure 4D, Supplementary Figure S4). Long-term depletion of CIT-K caused a continuous decrease in cell proliferation, as observed by an increased doubling time (T_d_) of CIT-K depleted cells, and after day 12 almost no proliferation occurred (Figure 4D, 4E). Measuring the fold difference between CIT-K depleted cells and control siRNA-treated cells revealed that, over time, the difference in proliferation increased dramatically, which correlates with increased multinucleation over time (Figure 4E, 3B).

**Figure 5:**
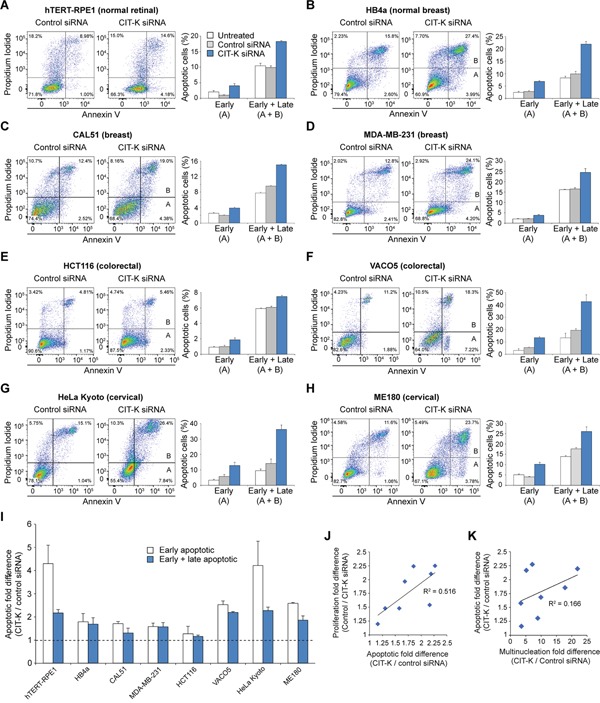
Cytokinesis failure induces apoptosis in cancer cells **A-H**. Cell lines were plated the day prior to transfection and were either untreated or treated with control or CIT-K siRNA for 72 h. Cells were harvested and stained with annexin V and propidium iodide (PI) to detect early apoptosis (annexin V positive, PI negative) and late apoptosis (annexin V positive, PI positive). Quantification of the flow cytometric data on the left is shown to the right, n=3. 20,000 cells were used in each analysis, error bars represent SEM. **I**. Quantification of fold difference between control and CIT-K-siRNA treated cells, n=3 (pooled data from A-H, n=9), error bars represent SEM. The dotted line represents a fold difference of 1; values above this line indicate an increase in apoptosis. **J**. Correlation analysis of proliferation and apoptotic fold differences. The values used for apoptotic fold difference is from the combined ‘Early + Late’ population. **K**. Correlation analysis of multinucleation and apoptotic fold differences. The Pearson correlation coefficients (R^2^) are listed next to the linear trend lines. The values used in J and K can be found in Table 1.

We then investigated the fate of CIT-K-depleted multinucleated cells. We measured cell death using an annexin V apoptotic assay combined with propidium iodide (PI) staining. Annexin V binds to phosphatidylserines that have flipped to the extracellular surface, identifying early apoptotic events in apoptosis, whereas combined PI staining marks late apoptotic events. Depleting CIT-K induced an apoptotic response, as indicated by an increase in both early apoptotic (annexin V positive, PI negative) and late apoptotic (annexin V positive, PI positive) cell populations (Figure 5A-5H). It is noteworthy that cell lines that either carry p53 mutations (MDA-MB-231 and VACO5) or have inactive p53 due to HPV infection (HeLa Kyoto and ME180) were still able to initiate apoptosis following CIT-K depletion (Figure 5D, 5F-5H), indicating that CIT-K depletion triggers cell death via p53-dependent and independent pathways. There was an increase in fold difference of both early and late apoptotic cell populations across all the cell lines tested (Figure 5I, Table 1), and we found a positive correlation (R^2^ = 0.516) between the level of proliferation decrease and the level of apoptosis (Figure 5J, Table 1) and a weak positive correlation (R^2^ = 0.166) between the increase of multinucleation and apoptosis after depletion of CIT-K (Figure 5J, Table 1).

**Figure 6:**
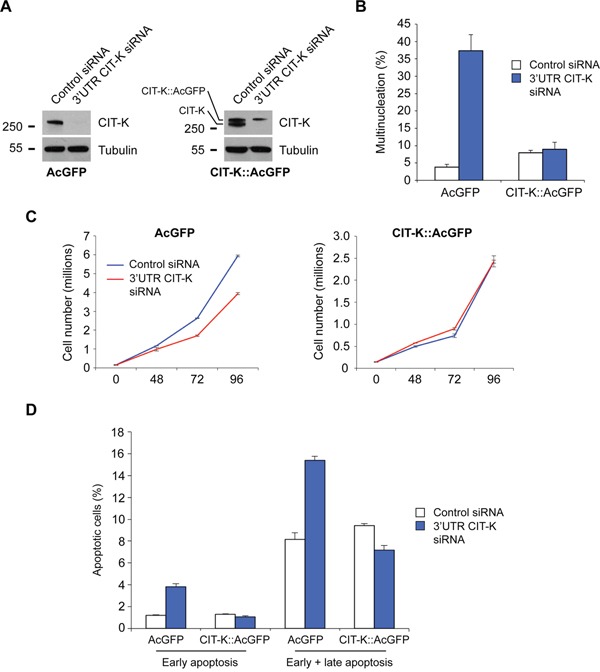
Exogenously expressed CIT-K rescues endogenously depleted CIT-K defects **A**. HeLa Kyoto cells stably expressing AcGFP or CIT-K::AcGFP were treated with either control or 3’UTR CIT-K siRNA for 48 h, and then cells were harvested and protein extracts analyzed by Western blot to detect CIT-K and tubulin. **B**. AcGFP and CIT-K::AcGFP cell lines were plated on glass coverslips and treated with either control or 3’UTR CIT-K siRNA for 48 h, and then cells were fixed and stained to detect tubulin and DNA by immunofluorescence microscopy. Over 600 cells were counted per condition, n=3, error bars represent SEM. **C**. AcGFP and CIT-K::AcGFP cell lines were treated with either control or 3’UTR CIT-K siRNA over a time course of 96 h, where at the respective time point the cell number was determined. Error bars represent SEM, n=3. **D**. AcGFP and CIT-K::AcGFP cell lines were treated with either control or 3’UTR CIT-K siRNA for 72 h, and then cells were harvested and stained with annexin V and PI to detect early and late apoptosis. Bar graph shows quantification of the flow cytometric data, n=4, 10,000 cells were used in each analysis, error bars represent SEM.

To confirm that the proliferation and apoptotic defects could be rescued by a siRNA-resistant CIT-K transgene, we generated monoclonal HeLa Kyoto cell lines stably expressing either human CIT-K tagged with AcGFP (*Aequorea coerulescens* GFP) or AcGFP alone. CIT-K::AcGFP correctly localized to the midbody ring like its endogenous counterpart (Supplementary Figure S5) [13] and was able to rescue cytokinesis failure, proliferation decrease and induction of apoptosis after depletion of endogenous CIT-K using an siRNA directed against the CIT-K 3’UTR, which is not present in the CIT-K::AcGFP transgene (Figure 6A-6D, Supplementary Figure S6). These results demonstrate that all these phenotypes are specific to CIT-K loss rather than off-target effects. Notably, cells over-expressing CIT-K::AcGFP proliferated much slower than control cells (Figure 6C), which is consistent with the published observation that cells over-expressing CIT-K take longer to complete abscission [15], and provides further evidence that CIT-K does not behave as an oncoprotein.

To conclude, our results indicate that cytokinesis failure caused by CIT-K depletion drastically inhibits cell proliferation and induces apoptosis in a variety of cancer cell lines.

### Both p53-dependent and independent pathways initiate apoptosis after cytokinesis failure

**Figure 7:**
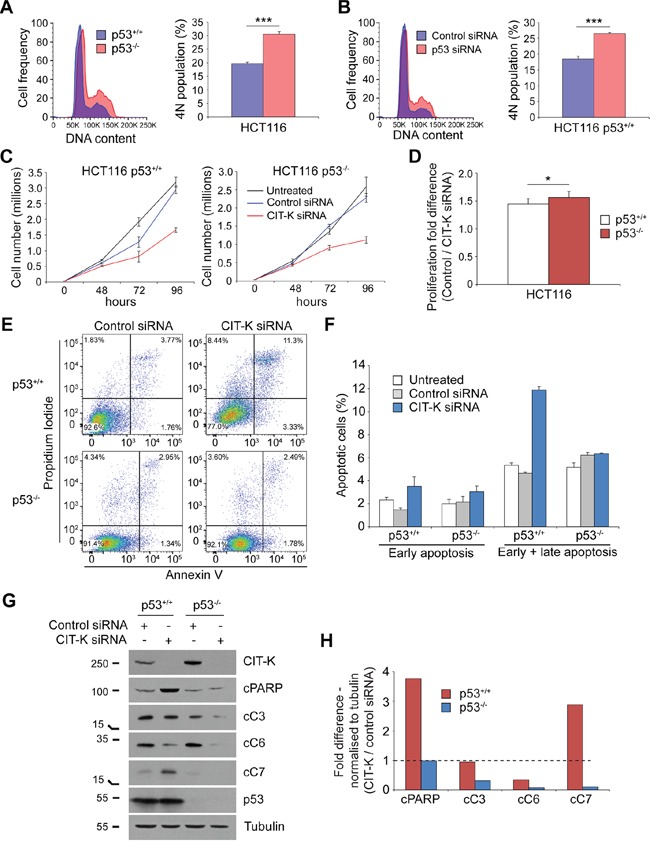
p53-dependent and independent pathways exist to initiate apoptosis following CIT-K depletion **A**. HCT116 p53^+/+^ and p53^-/-^ cells were fixed in 70% ethanol and treated with PI/RNase solution to detect DNA content. 4N DNA content was analyzed by gating for the second, G2/M peak. 20,000 cells were used for each analysis, n=7, ***p < 0.001 (Student's *t*-test), error bars represent SEM. **B**. HCT116 p53^+/+^ cells were treated with either control or TP53 siRNA for 48 h, harvested, fixed in 70% ethanol and treated with PI/RNase solution to detect DNA content. 4N DNA content was analyzed by gating for the second, G2/M peak. 20,000 cells were used for each analysis, n=6, ***p < 0.001 (Student's *t*-test), error bars represent SEM. **C**. HCT116 p53^+/+^ and p53^-/-^ cells were plated prior to transfection and were either untreated or treated with control or CIT-K siRNA at day 0 for up to 96 h. Cell number was calculated at each time point, each condition was performed in triplicate, error bars represent SEM. **D**. Quantification of fold difference between control and CIT-K-siRNA treated cells at 96 h, n=6 (pooled data from C, n=18), *p < 0.05 (Student's *t*-test), error bars represent SEM. **E**. HCT116 p53^+/+^ and p53^-/-^ cells were treated with either control or CIT-K siRNA for 72 h, where cells were harvested and stained with annexin V and PI to detect apoptosis. 20,000 cells were used in each analysis. **F**. Quantification of the flow cytometric data shown in E. Errors bars represent SEM, n=3. **G**. HCT116 p53^+/+^ and p53^-/-^ cells were treated with either control or CIT-K siRNA for 48 h, harvested and protein extracts analyzed by Western blot to detect CIT-K, cleaved PARP, cleaved caspases 3, 6 and 7, p53 and tubulin. The numbers on the left indicate the sizes in kilodaltons of the molecular mass marker. **H**. Band intensities of the Western blot analysis from G was analyzed by ImageJ, normalized to tubulin, and the fold difference between CIT-K and control siRNA lysates calculated. The dotted line represents a fold difference of 1; values above this line indicate an increase of protein cleavage and thus activation.

It is well known that p53 is one of the main factors that can induce apoptosis in response to various stress signals [35]. However, we observed an increase in apoptosis after CIT-K depletion in cancer cells with impaired p53 functions (Figure 5D, 5F-5H). To investigate the role of p53 in cell death induced by cytokinesis failure, we used two isogenic strains of the colon carcinoma cell line HCT116: a parental p53^+/+^ cell line and a mutated p53^-/-^ cell line [36]. We first analyzed the DNA content of both lines and found an increased amount of cells containing 4N in the p53^-/-^ cell population when compared to p53^+/+^ cells (Figure 7A). This was recapitulated by transiently knocking down p53 in HCT116 p53^+/+^ cells (Figure 7B). Interestingly, a greater level of multinucleation was observed in p53^-/-^ cells (7.97%) compared to p53^+/+^ cells (2.21%) under control conditions (Supplementary Figure S7A, S7B), indicating, as expected, that p53 is necessary to prevent multinucleation. We found that loss of p53 affected cell proliferation after CIT-K depletion, as HCT116 p53^-/-^ cells showed a slight, but significant, reduction in their proliferation compared to p53^+/+^ cells (Figure 7C, 7D, Supplementary Figure S7A, S7B). Interestingly, although p53^-/-^ cells divided more slowly after CIT-K depletion, they did not appear to initiate apoptosis (Figure 7E, 7F). CIT-K depletion in HCT116 p53^+/+^ cells caused an increase in cleaved executioner caspase 7 (cC7) and its downstream target poly(ADP-ribose) polymerase (cPARP), but not executioner caspases 3 (cC3) and 6 (cC6) (Figure 7G, 7H), indicating that, in these cells, cytokinesis failure triggers apoptosis primarily via caspase 7 activation. By contrast, depleting CIT-K in p53^-/-^ cells did not increase cleavage of PARP or any major executioner caspases (Figure 7G, 7H), consistent with the well-known pro-apoptotic role of p53.

Interestingly, the HCT116 p53^-/-^ cells exhibited an increase of CIT-K expression (Figure 7G). To determine whether this is a real effect due to loss of p53 or an artifact, we transiently depleted p53 over a time course of 96 hours and found that CIT-K was significantly upregulated compared to control conditions (Supplementary Figure S8A, S8B). Together, this suggests that p53 could somehow regulate the expression of CIT-K, potentially functioning in an inhibitory manner.

**Figure 8:**
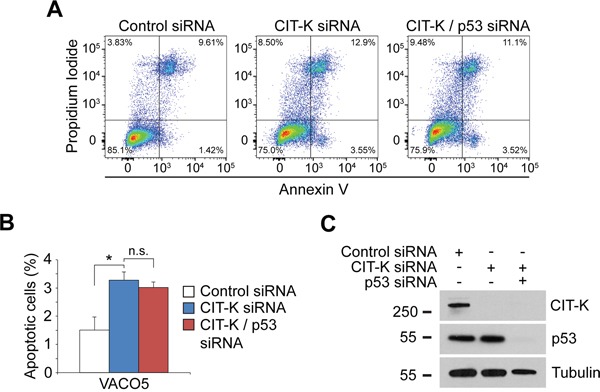
VACO5 colorectal cells still undergo apoptosis when co-depleted for CIT-K and p53 **A**. VACO5 cells were treated with control, CIT-K or combination CIT-K/TP53 siRNA for 72 h, where cells were harvested and stained with annexin V and PI to detect apoptosis. 50,000 cells were used in each analysis. **B**. Quantification of the early apoptotic fraction (annexin V positive, PI negative). Error bars represent SEM, n=4, *p < 0.05 (Student's *t*-test), n.s., not significant. **C**. VACO5 cells were treated with control, CIT-K or combination CIT-K/TP53 siRNA for 72 h, where cells were harvested and protein extracts analyzed by Western blot to detect CIT-K, p53 and tubulin. The numbers on the left indicate the sizes in kilodaltons of the molecular mass marker.

The finding that p53 is required for apoptosis following cytokinesis failure in HCT116 cells is in contrast with our observation that other p53-defective cancer cell lines were able to initiate apoptosis after depletion of CIT-K (Figure 5D, 5F-5H). To eliminate the possibility that these cell lines could still possess some residual p53 apoptotic functions, we measured apoptosis after co-depletion of CIT-K and p53 in p53-mutated VACO5 colorectal cancer cells. No significant difference in apoptosis was observed between VACO5 cells treated either with CIT-K siRNA alone or with both CIT-K and p53 siRNAs (Figure 8A-8C), indicating that p53-mutated cells undergo cell death via a p53-independent apoptotic mechanism after depletion of CIT-K. In conclusion, these results demonstrate that cytokinesis failure triggers apoptosis via both p53-dependent and independent pathways.

### Tetraploid and actively dividing cells are more sensitive to cytokinesis failure

**Figure 9:**
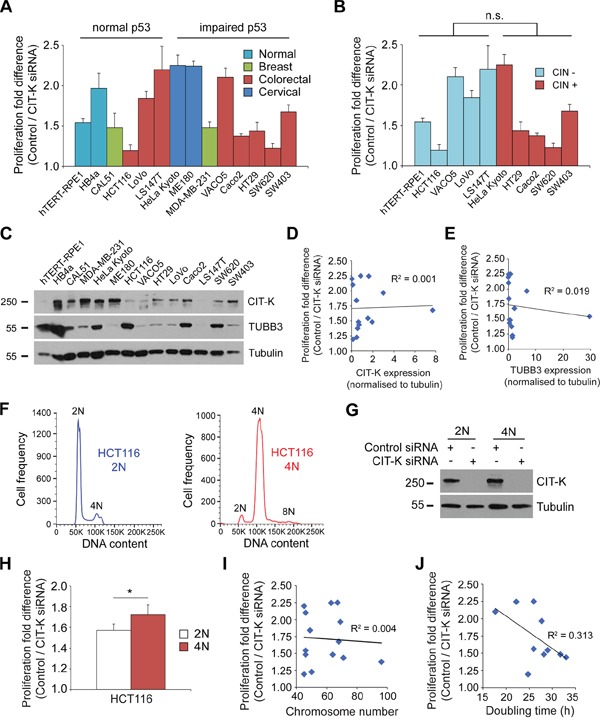
Tetraploid cells are more sensitive to the depletion of CIT-K compared to their diploid counterparts **A**. Cell lines listed were treated with control or CIT-K siRNA for 96 h, cell number calculated and the fold difference between control and CIT-K siRNA-treated cells quantified. For each condition n=3, with the experiment performed in triplicate (pooled data, n=9), error bars represent SEM. The cell lines are sorted into having normal or impaired p53 functions. **B**. Cells listed were treated as described in A, but were characterized depending on whether they possessed CIN (+) or not (-). **C**. Protein extracts of all the cell lines tested were analyzed by Western blot to detect CIT-K, TUBB3 and tubulin. The numbers on the left indicate the sizes in kilodaltons of the molecular mass marker. **D**. Correlation analysis of proliferation fold difference and CIT-K expression (from C, CIT-K normalized to tubulin) of each cell line. The Pearson correlation coefficient (R^2^) is listed next to the linear trend line. **E**. Correlation analysis of proliferation fold difference and TUBB3 expression (from C, CIT-K normalized to tubulin) of each cell line. **F**. HCT116 2N and 4N cells were fixed in 70% ethanol and treated with PI/RNase solution to detect DNA content. 50,000 cells were used for each analysis. **G**. HCT116 2N and 4N cells were treated with either control or CIT-K siRNA for 48 h, harvested and protein extracts analyzed by Western blot to detect CIT-K and tubulin. The numbers on the left indicate the sizes in kilodaltons of the molecular mass marker. **H**. HCT116 2N and 4N cells were treated with either control or CIT-K siRNA for 48 h, counted and the fold difference between control and CIT-K siRNA-treated cells quantified. For each condition n=9, *p < 0.05 (Student's *t*-test), error bars represent SEM. **I**. Correlation analysis of proliferation fold difference and chromosome number of each cell line. **J**. Correlation analysis of proliferation fold difference and doubling time of cell lines. The values used for D, E, I and J can be found in Table 1.

Comparative analysis of cell proliferation after CIT-K depletion in eight different cell lines (Figure 4A, 4B) indicated that the cells that had the largest decreases in proliferation were VACO5, HeLa Kyoto and ME180, all of which have impaired p53 functions (Table 1). Therefore, we reasoned that inactivation of p53 could potentially render cells more susceptible to cytokinesis failure, which was also consistent with our finding that HCT116 p53^-/-^ cells proliferated slower than their parental p53^+/+^ cells after CIT-K depletion (Figure 7C, 7D). To address this, we extended our panel of colorectal cell lines to include additional lines harboring either wild type (LoVo and LS147T) [33] or mutated p53 genes (Caco2, HT29, SW620 and SW403) [33, 37, 38] (Table 1). Western blot analysis confirmed a severe knockdown of CIT-K in all six additional cell lines (Supplementary Figure S9). However, contrary to our expectation, the p53 status did not appear to be a major determining factor in the susceptibility of cancer cells to cytokinesis failure (Figure 9A, Table 1). Similarly, CIN status also did not seem to be a contributing factor (Figure 9B, Table 1).

We then investigated whether CIT-K expression levels could determine how cells responded to CIT-K knockdown, but found no significant correlation (R^2^ = 0.001) between the level of CIT-K expression and proliferation decrease (Figure 9C, 9D, Table 1), consistent with the finding that over-expression of CIT-K does not possess oncogenic potential (Figure 2A and 2B). It was recently reported that tubulin β-III (TUBB3) over-expression increased sensitivity to CIT-K depletion in HeLa cells [39]. However, our analysis indicated no correlation (R^2^ = 0.019) between the level of TUBB3 expression and how cells responded to CIT-K knockdown (Figure 9C, 9E, Table 1).

Our findings indicated that high levels of multinucleation induced by long CIT-K RNAi treatments resulted in large decreases in cell proliferation (Figures 3B, 4D), suggesting that an increase in ploidy might render cells more sensitive to cytokinesis failure. To address this hypothesis, we generated a tetraploid HCT116 cell line by inducing a single round of cytokinesis failure using the actin depolymerizing drug cytochalasin D (Figure 9F) and compared how these cells responded to CIT-K depletion with respect to their parental diploid counterparts. Western blot analysis indicated that there was no difference in the level of CIT-K expression and CIT-K RNAi knockdown efficiency between the two cell lines (Figure 9G). We found that tetraploid HCT116 cells were more sensitive to cytokinesis failure than diploid cells, as shown by a significant difference in cell proliferation (Figure 9H, Table 1). However, when we plotted the proliferation fold difference against the chromosome numbers of all the cell lines used in this study, no direct correlation (R^2^ = 0.004) was observed (Figure 9I). We did, instead, find a modest positive correlation (R^2^ = 0.313) between the cell cycle rate and the decrease in cell proliferation (Figure 9J), suggesting that fast dividing cells are more susceptible to cytokinesis failure.

Taken together, all these results indicate that CIT-K depletion was particularly effective in blocking the proliferation of polyploid and actively dividing cells, irrespective of the level of CIT-K expression, p53 status and CIN.

## DISCUSSION

The discovery and validation of new targets for the treatment of cancers is essential for improving patient survival. Here we investigated the potential of inducing cytokinesis failure via depletion of CIT-K as an anti-proliferative anti-cancer approach in breast, cervical and colorectal cancers. Our results indicate that CIT-K is essential for cytokinesis in all cancer cell lines, which contrasts with the evidence that in CIT-K null mice cytokinesis failure was only observed in brains and testes [28, 29]. It is therefore likely that CIT-K is necessary for cytokinesis across a wider range of human tissues than initially anticipated from studies in mice. These findings also suggest that, notwithstanding the high level of sequence conservation between mice and human genes, their functions and requirement can differ in the two organisms and caution should be taken in assuming that knowledge gained from studies in mice could be directly transferred to human systems.

The Hippo tumor suppressor pathway was activated after CIT-K depletion through phosphorylation of LATS2 in non-cancerous immortalized hTERT-RPE1 cells (Figure 3D, left panel), which acts to limit cell proliferation by negatively regulating growth transcriptional co-activators YAP and TAZ and by stabilizing p53 [34]. The Hippo pathway is well known to regulate apoptosis to control organ size and to respond to DNA damage [40–43], but whether it can regulate apoptosis in response to tetraploidization is not clear. The Hippo tumor suppressor pathway has been shown to be deregulated in a broad range of cancers, contributing towards tumorigenesis [44]. In support of this, we show that HeLa Kyoto and HCT116 cancer cells did not activate the Hippo pathway after cytokinesis failure (Figure 3D, middle and right panels), suggesting that these cancer cells have evolved alternative pathways to detect cytokinesis failure (see also next paragraphs).

CIT-K depletion induced apoptosis through executioner caspase 7 and PARP (Figure 7G, 7H). Interestingly, executioner caspases 3 and 6 were not activated, which is not surprising as this caspase network is known to possess distinct, non-redundant functions [45–47]. Loss of p53 also causes polyploidy (Figure 7A, 7B) [48–50], which in turn can render cells more susceptible to CIT-K depletion (Figure 7C, 7D). In addition, our results suggest that cytokinesis failure can trigger apoptosis via distinct mechanisms in cancer cells (Figures 5, 7, 8). We found that p53 was essential for initiating apoptosis following cytokinesis failure in HCT116 cells (Figure 7E-7H), as previously reported [51, 52]. However, cells with impaired p53 functions (i.e. MDA-MB-231, VACO5, HeLa Kyoto and ME180) were still able to initiate apoptosis (Figure 5A-5I), even after p53 RNAi (Figure 8A-8C), suggesting that these cells have acquired p53-independent mechanisms to trigger apoptosis. It is unclear why there is this apoptotic discrepancy between the isogenic HCT116 strains and the p53-defective lines, but one possibility is that it could be a difference in how cells have evolved in the complete absence versus just mutation of p53.

What could be the apoptosis-trigger mechanism in p53-deficient cells? The cyclin-dependent kinase inhibitor p21 is activated by p53 to inhibit S phase entry to allow the cell time to repair any potential DNA damage. However, p21 can be activated independently of p53 to promote apoptosis [53], and it is therefore conceivable that p21 could be responsible for regulating/initiating apoptosis in response to CIT-K depletion in cells harboring p53 mutations. Indeed, Chk2 has been shown to activate p21 independently of p53 leading to cell cycle arrest, apoptosis and senescence [54]. Importantly, these results, along with the lack of correlation between the p53 status and susceptibility to CIT-K depletion (Figure 9A), indicate that cytokinesis failure could be an effective anti-proliferative approach for treating cancers carrying either wild type or mutated p53. This evidence, along with our findings that CIT-K depletion inhibited proliferation in all cancer cell lines and the studies mentioned earlier in prostate and hepatocellular cancer cells [19, 20], suggest that targeting CIT-K might be a potentially valid therapeutic approach for the treatment of various cancer types. However, our study also indicates that CIT-K depletion was more effective in actively dividing and, in some cases, polyploid cancer cells (Figure 9A, 9H and 9J), which suggests that inhibiting cytokinesis might be an effective strategy for the treatment of fast proliferating, Myc-driven cancers. Additional *in vivo* studies, however, are necessary to test these hypotheses.

CIT-K's kinase activity is required for completion of cytokinesis [12, 13, 55] and this kinase has been shown to phosphorylate itself and the INCENP component of the chromosomal passenger complex [13]. Together, these findings would indicate that the isolation of small molecules able to interfere with the ATP binding pocket of CIT-K could be a feasible objective and these inhibitors might become valid anti-proliferative tools in cancer therapy. In addition, as mentioned earlier, CIT-K is an attractive target because it is the only kinase to function solely in cytokinesis – in contrast to other kinases such as Aurora B and Plk1, which possess pleiotropic roles throughout mitosis – and thus it is conceivable that targeting CIT-K could cause less undesired side effects.

Finally, it is important to consider that targeting cytokinesis as a means of cancer therapy is a double-edged sword approach. On the one hand, it is effective in inducing cell death, on the other, cytokinesis failure and polyploidy have been shown to promote tumorigenesis, CIN and drug resistance [7–9, 56, 57]. However, tumorigenesis is only promoted in tetraploid cells devoid of p53, implying that, if healthy cells were subject to cytokinesis failure, p53 would limit CIN [7]. Therefore, although it seems likely that long treatments with cytokinesis inhibitors may quash potential cancer-generating tetraploid cells, caution has to be taken when investigating the use of cytokinesis inhibitors in cancer therapy.

## MATERIALS AND METHODS

### Cell lines

All cells were maintained at 37°C, 5% CO_2_. hTERT-RPE1, CAL51, MDA-MB-231, HCT116 (all variants), VACO5, HT29, LoVo, LS147T, SW620, Caco2 and SW403 cells were cultured in DMEM/F12, 2mM L-glutamine and 10% FBS. HeLa Kyoto cells (wild type, AcGFP and CIT-K::AcGFP stable cell lines) were cultured in DMEM GlutaMAX and 10% FBS. HB4a cells were cultured in RPMI-1640, 5 μg/ml hydrocortisone, 5 μg/ml insulin and 10% FBS. ME180 cells were cultured in GMEM, 2mM L-glutamine and 10% FBS. NIH3T3 cells were cultured in DMEM and 10% NCS. Generation of the AcGFP and CIT-K::AcGFP cell line was performed as previously described [13], however we used the pIRESpuro3 vector (Clontech) with Flag and AcGFP C-terminal tags (for simplicity this construct was referred to as AcGFP).

### Antibodies

The following antibodies were used: mouse monoclonal anti-α-tubulin (clone DM1A, Sigma, T9026), mouse monoclonal anti-CIT-K (BD Transduction Laboratories, 611377), rabbit anti-cleaved caspase 3 (Cell Signaling Technology, CST, 9661), rabbit anti-cleaved caspase 6 (CST, 9761), rabbit anti-cleaved caspase 7 (CST, 9491), rabbit anti-cleaved PARP (CST, 9541), mouse monoclonal anti-p53 (clone D0-1, Santa Cruz Biotechnology, sc-126), rabbit anti-phosphorylated LATS (CST, D57D3), rabbit anti-LATS2 (Bethyl Laboratories, A300-479A) and mouse monoclonal anti-TUBB3 (clone 2G10, Abcam, ab78078). Peroxidase and Alexa-fluor conjugated secondary antibodies were purchased from Jackson Laboratories and Invitrogen respectively.

### Colony formation assay

NIH3T3 cells were plated at a density of 1×10^5^ per 6cm dish and were transfected with 1 μg of respective DNA using FuGENE HD Transfection Reagent (Promega) according to manufacturer's instructions, at a FuGENE HD:DNA ratio of 3:1 for total DNA transfected. Media was changed every 4 days for 2 weeks, where cells were then fixed in PHEM buffer (60 mM PIPES, 25 mM HEPES pH 7, 10 mM EGTA, 4 mM MgCl_2_, 3.7% [v/v] formaldehyde) for 15 minutes and stained with crystal violet solution (0.5% [w/v] crystal violet and 25 mM HEPES) for 60 minutes. Crystal violet solution was washed off with Milli-Q water. Colonies above 2 mm in diameter were counted for analysis. The following plasmids were used: pBABE empty vector, pBABE::hK-rasV12, pIRESpuro3::AcGFP empty vector, pIRESpuro3::CIT-K::AcGFP, Venus empty vector and Aurora A::Venus (kind gifts of Dr. C. Lindon, University of Cambridge).

### siRNA transfection

The following siRNAs were used: scrambled sequence control – 5’-AACGUACGCGGAAUACUUCGA-3’, CIT-K – 5’-AUGGAAGGCACUAUUUCUCAA-3’, 3’UTR CIT-K – 5’-CACACUAUGGAACUCUGCU-3’, TP53 – a pool of siRNAs were purchased from Dharmacon. All siRNAs were transfected at a concentration of 12 nM using lipofectamine RNAiMAX (Thermo Fisher Scientific) following the manufacturer's instructions, where cells were then washed with PBS and maintained under normal culture conditions for the specified time.

### Immunofluorescence multinucleation assay

Cells were grown on microscope glass coverslips (Menzel-Gläser), treated with respective siRNAs for the specified time and fixed in PHEM buffer for 12 min. They were then washed three times for 10 min with PBS and incubated in blocking buffer (PBS, 0.5% [v/v] Triton X-100 and 1% [w/v] BSA) for 1 hour (h) at room temperature (RT). Coverslips were incubated overnight at 4°C with the primary antibodies indicated in the figure legends, diluted in PBT (PBS, 0.1% [v/v] Triton X-100 and 1% [w/v] BSA). The following day, coverslips were washed twice for 5 min in PBT, incubated with secondary antibodies diluted in PBT for 2 h at RT and then washed twice with PBT and once with PBS. Coverslips were mounted on SuperFrost Microscope Slides (VWR) using VECTASHIELD Mounting Medium containing DAPI (Vector Laboratories). Over 600 cells per condition were counted for analysis.

### Proliferation assay

Cell proliferation was measured by counting absolute cell number. Cells were treated with respective siRNAs for the specified time, harvested by trypsinisation, an aliquot of cells was stained with an equal volume of 0.4% [w/v] trypan blue (Sigma Aldrich), pipetted into disposable Countess^®^ chamber slides and counted using the Countess^®^ Automated Cell Counter (Thermo Fisher Scientific). Only viable cells were measured in the analysis. Doubling time (T_d_) of cells was calculated with the following formula: T_d_ = duration of analysis (hours) / (log(final cell number / initial cell number) / log(2)).

### Apoptosis assay

Cells were treated with respective siRNAs for 72 hours, harvested using non-enzyme dissociation buffer (Sigma Aldrich) and kept on ice. The media and PBS wash prior to harvesting were collected as apoptotic cells can become buoyant. Dissociated cells were carefully collected, washed twice in ice cold PBS and once in 1X annexin V binding buffer (BD Pharmingen). Approximately 1×10^5^ cells were stained with annexin V-FITC (BD Pharmingen) for 15 minutes in the dark at RT and then with PI (BD Pharmingen) just prior to analysis on the Cytek FACScan™ Flow Cytometer. 20,000 cells were used in the analysis.

### Cell cycle analysis

Cells were harvested, washed with PBS and fixed for 30 minutes at 4°C with ice cold 70% [v/v] ethanol (adding ethanol drop wise to cells whilst vortexing). Fixed cells were washed with PBS and re-suspended in FxCycle™ PI/RNase Staining Solution (Life technologies) for 15 minutes prior to analysis on the Cytek FACScan™ Flow Cytometer. 50,000 cells were used in the analysis, unless stated otherwise.

### Generation of tetraploid HCT116 cell line

HCT116 cells (the HCT116 p53^+/+^ strain was used) were treated with 4 μM cytochalasin D (Sigma Aldrich) for ∼18 hours, washed every 5 minutes for 30 minutes with PBS, stained with 15 μM Hoescht 33342 for 30 minutes at 37°C and then FACS sorted for the 8N population (to isolate dividing 4N cells). Cells were grown until near confluent in a T75 flask where they were again sorted for the 8N population (no treatment with cytochalasin D on subsequent sorts). This process was repeated twice more until a near pure population of dividing HCT116 4N cells were achieved. For the parental 2N cell line, cells were sorted for the 2N population following cytochalasin D treatment and cultured under normal conditions.

### Oncomine™ analysis

The following datasets were used to analyse *CIT* mRNA expression in cancers compared to healthy tissue. Where the dataset ID was not available (typically prefaced by GSE), the PubMed ID (PMID) was given. *Bladder*: GSE13507, GSE3167, GSE89, PMID 16432078. *Brain*: GSE2223, GSE4290, GSE4058, GSE4536, GSE7696, TCGA, PMID 12894235, PMID 11929829, PMID 16357140. *Breast*: GSE3744, GSE3193, GSE4382, GSE5764, GSE22358, GSE1477, GSE3971, GSE14548, GSE8977, GSE9014, TCGA, PMID 10963602, PMID 22522925. *Cervical*: GSE7410, GSE6791, GSE9750, GSE7803. *Colorectal*: GSE20916, GSE9689, GSE20842, GSE9348, GSE20916, GSE8671, GSE5206, GSE6988, TCGA, PMID 12101425. *Esophageal*: GSE20347, GSE6059, GSE23400, GSE13898, GSE1420. *Gastric*: GSE27342, GSE13861, GSE13911, GSE19826, PMID 12925757. *Head and neck*: GSE27155, GSE25099, GSE6631, GSE13601, GSE1722, GSE3524, GSE2379, GSE6791, GSE9844, GSE6004, GSE3467, GSE12452, PMID 12368205, PMID 15833835, PMID 14729608. *Kidney*: GSE15641, GSE4125, GSE11151, GSE2712, GSE14994, GSE6344, GSE781. *Leukaemia*: GSE2350, GSE13159, GSE2466, GSE7186, GSE1159, GSE28497, GSE1466, GSE995, GSE5788, PMID 16267031. *Liver*: GSE6764, GSE3500, GSE14520, GSE14323. *Lung*: GSE2514, GSE7670, GSE31210, GSE10072, GSE19188, GSE32863, GSE3398, GSE3268, PMID 11707567, PMID 15833835. *Lymphoma*: GSE6338, GSE2350, GSE3827, GSE1466, GSE12195, GSE14879, GSE12453. *Melanoma*: GSE7553, GSE3189, GSE6887, PMID 15833814. *Myeloma*: GSE13591, GSE5900. *Ovarian cancer*: GSE6008, GSE26712, GSE12470, TCGA, PMID 14760385, PMID 15161682. *Pancreatic*: GSE16515, GSE15471, GSE3654, GSE1542, PMID 16103885, PMID 15867264, PMID 15548371. *Prostate*: E-TABM-26, GSE6099, GSE21034, GSE6956, GSE6919, GSE3325, GSE3933, PMID 12873976, PMID 19737960, PMID 22722839, PMID14695335, PMID 11807955, PMID 12086878, PMID 11507037, PMID 12154061. *Sarcoma*: GSE21122, GSE13861, GSE2719, GSE2712.

## SUPPLEMENTARY FIGURES


