# Supplementary material for: Investigating cytokinesis failure as a strategy in cancer therapy
Source: Oncotarget. 2016 Nov 24;7(52):87323–41. doi: 10.18632/oncotarget.13556 (PMC5349991; doi:10.18632/oncotarget.13556)
Supplement: Supplementary file 1 [file oncotarget-07-87323-s001.pdf]

# Investigating cytokinesis failure as a strategy in cancer therapy

## SUPPLEMENTARY FIGURES

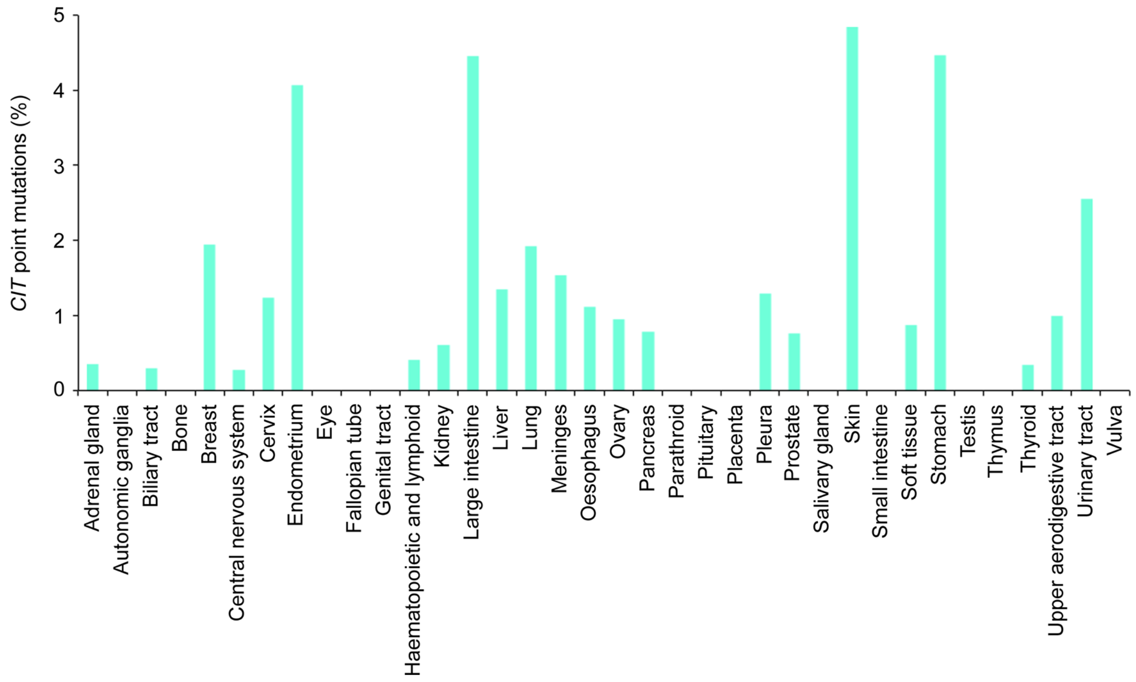


**Supplementary Figure S1: *CIT* is mutated in a low percentage of cancers from a variety of tissues.** Graph showing the *CIT* point mutations identified from the COSMIC database (gene ID: CIT_ENST00000261833).

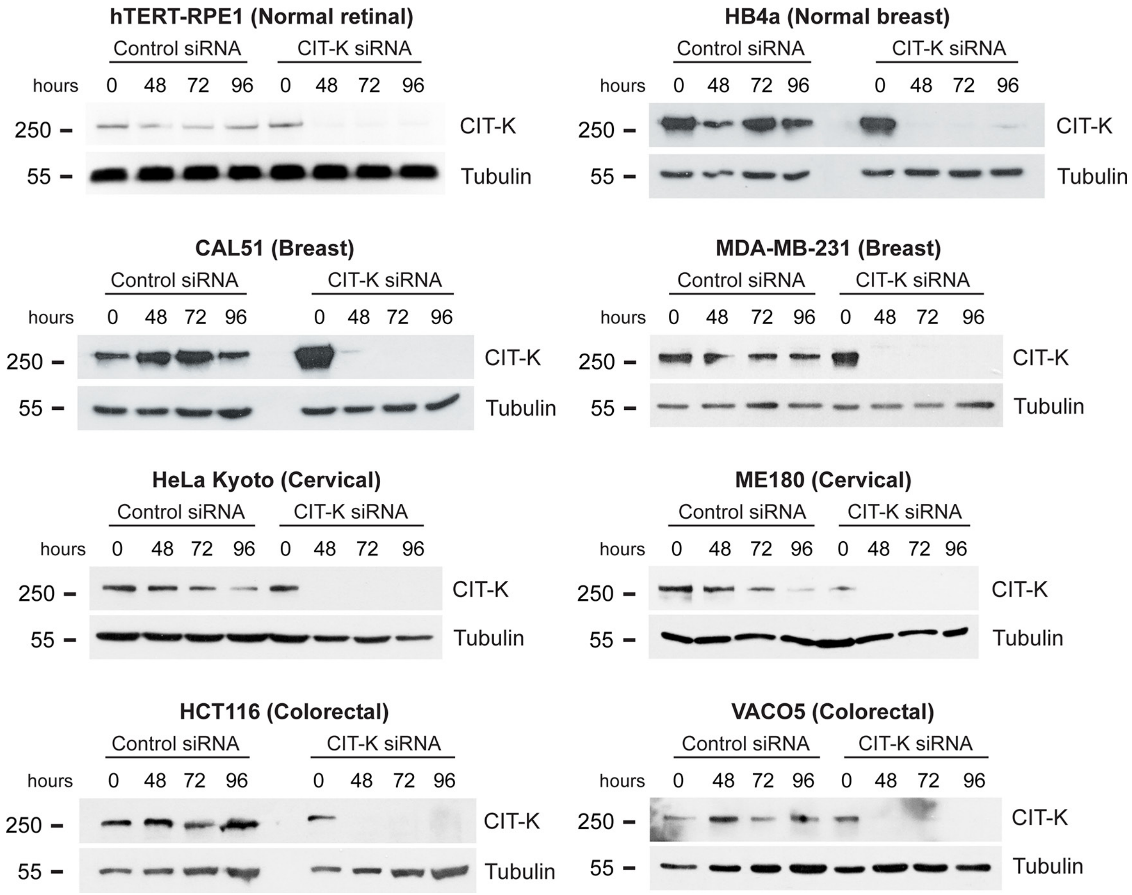


**Supplementary Figure S2: Knockdown of CIT-K via RNAi in hTERT-RPE1, HB4a, CAL51, MDA-MB-231, HCT116, VACO5, HeLa Kyoto and ME180 cell lines.** Cells were plated prior to transfection and treated with either control or CIT-K siRNA for 48, 72 or 96 h. Aliquots of cells were harvested at each time point and protein extracts analyzed by Western blot to detect CIT-K and tubulin. The numbers on the left indicate the sizes in kilodaltons of the molecular mass marker.

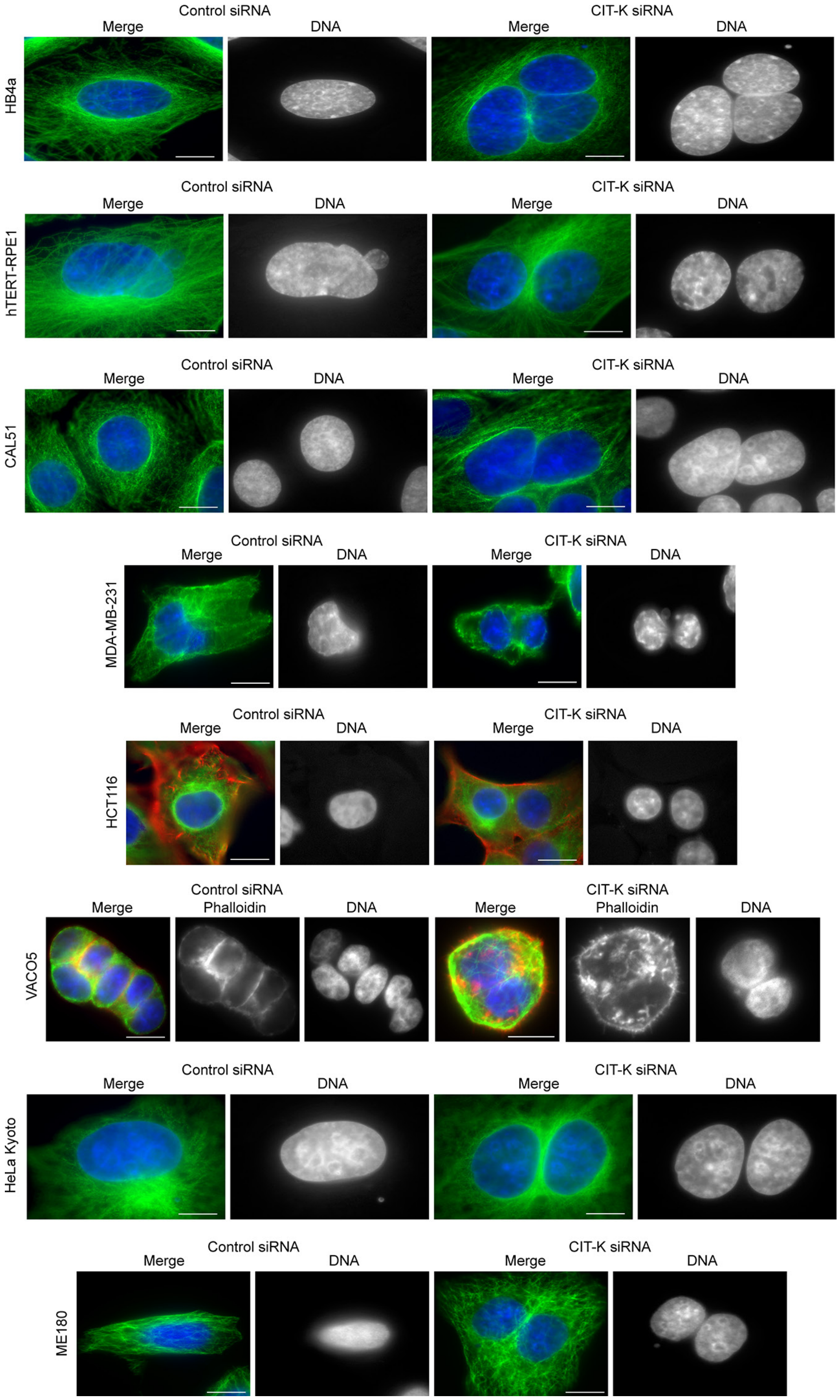


**Supplementary Figure S3: Depleting CIT-K fails cytokinesis in breast, colorectal and cervical cell lines.** Cells were plated on coverslips prior to transfection and treated with either control or CIT-K siRNA for 48 h, where cells were fixed and stained to detect tubulin (green), phalloidin (if used, red) and DNA (blue). Bars, 10 µm.

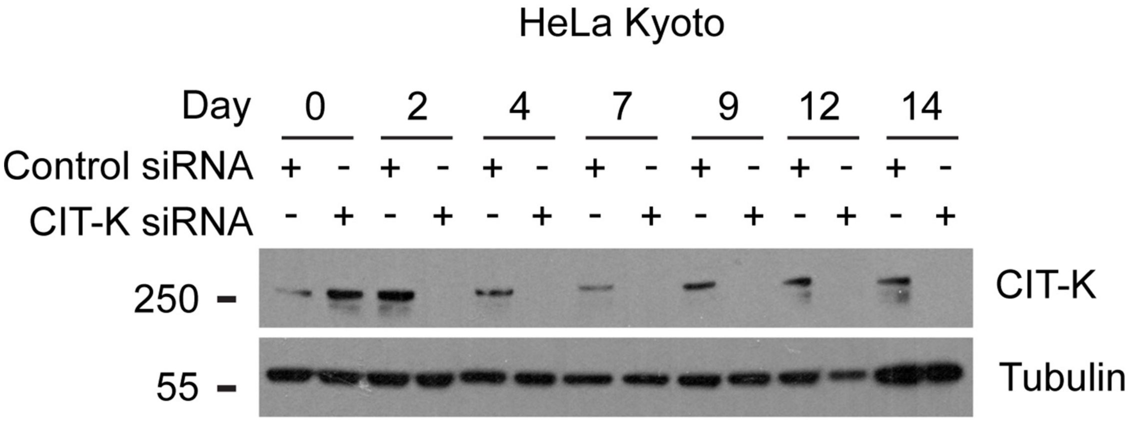


**Supplementary Figure S4: Long-term, repeated knockdown of CIT-K via RNAi.** HeLa Kyoto cells were plated prior to transfection and treated with either control or CIT-K siRNA for 48 and 96 h, where cells were harvested at each time point and protein extracts analyzed by Western blot to detect CIT-K and tubulin. At 96 h, both control and CIT-K siRNA-treated cells were re-plated and transfected for two additional rounds to extend the time point to 14 days. The numbers on the left indicate the sizes in kilodaltons of the molecular mass marker.

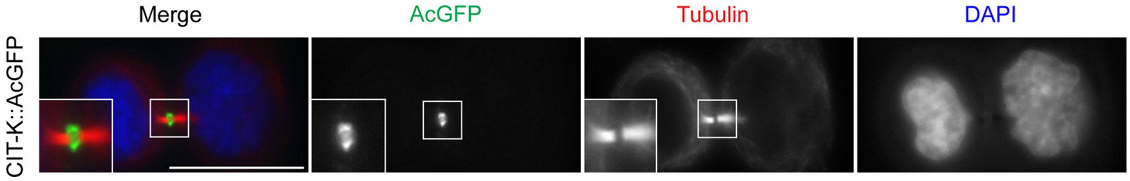


**Supplementary Figure S5: CIT-K::AcGFP localizes correctly to the midbody ring.** HeLa Kyoto cells stably expressing CIT-K::AcGFP were stained for AcGFP (green), tubulin (red) and DNA (blue). Scale bar, 20 µm. Inset shows a 2X magnification of the midbody.

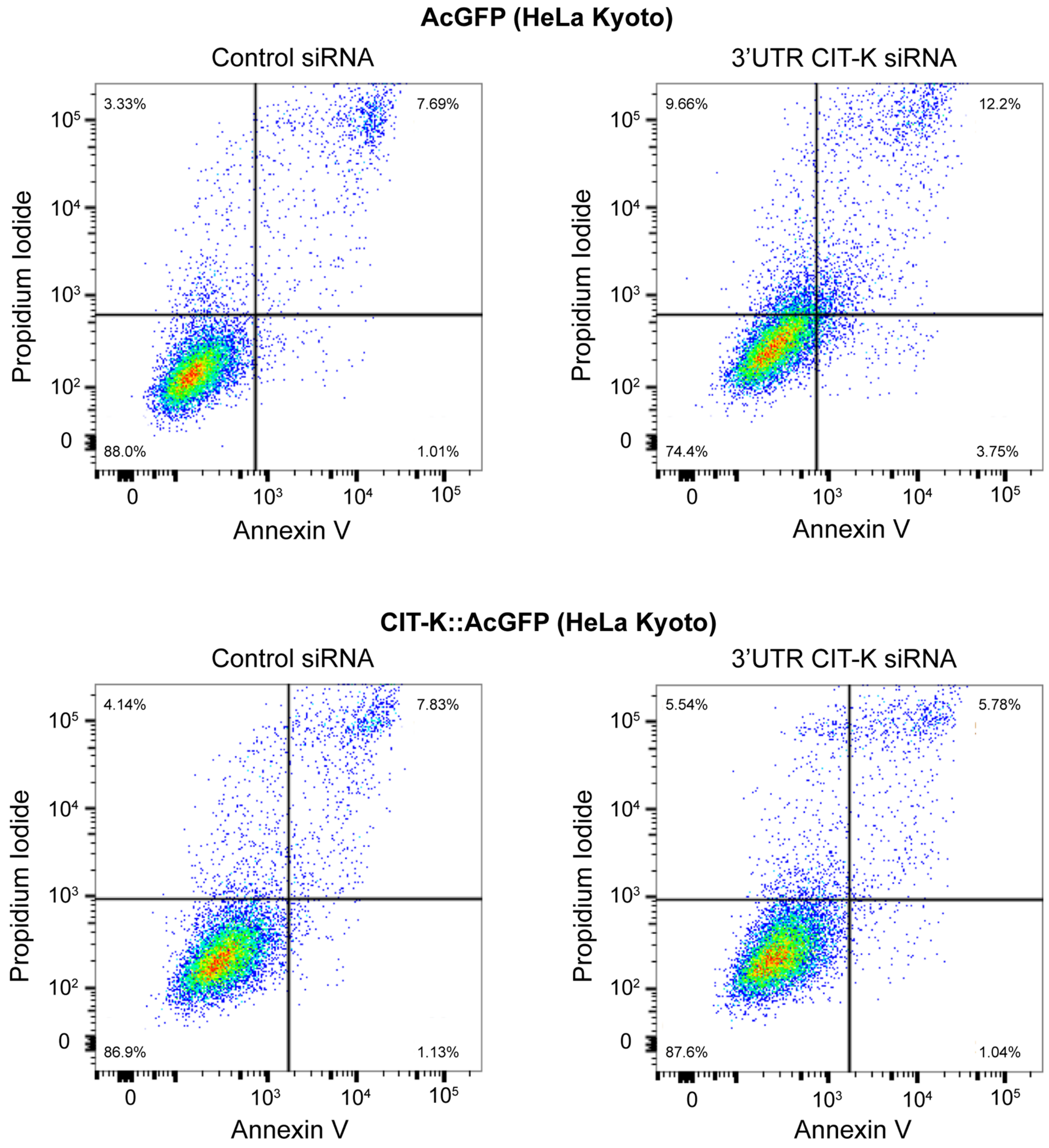


**Supplementary Figure S6: CIT-K::AcGFP rescues cells initiating apoptosis after endogenous CIT-K depletion.** HeLa Kyoto cells stably expressing either AcGFP or CIT-K::AcGFP were treated with either a control or 3'UTR CIT-K siRNA for 72 h, where cells were harvested and stained with annexin V and PI to detect early and late apoptosis. Bar graph shows quantification of the flow cytometric data, n=4, 10,000 cells were used in each analysis, error bars represent SEM.

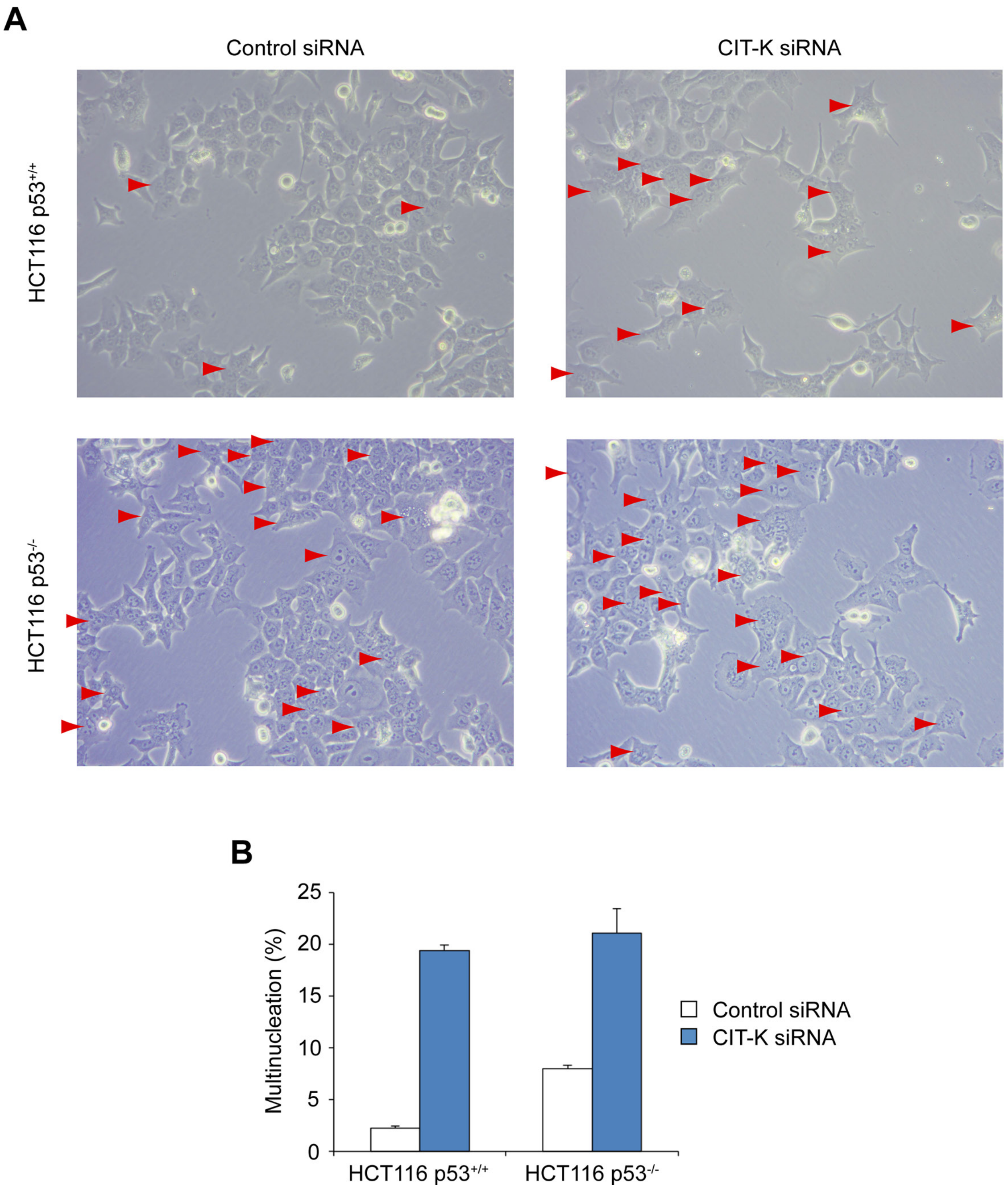


**Supplementary Figure S7: HCT116 p53$^{+/+}$ and p53$^{-/-}$ cell lines fail cytokinesis following CIT-K depletion. A.** HCT116 p53$^{+/+}$ and p53$^{-/-}$ cells were treated with either control or CIT-K siRNA for 48 h, where cells were imaged using a light microscope. Red arrows indicate multinucleated cells. **B.** Quantification of the images shown in A. All cells in view were counted and the percentage of multinucleated cells calculated. At least 400 cells were counted for each experiment (multiple screen shots used per analysis), with the experiment repeated in triplicate. Error bars represent SEM.

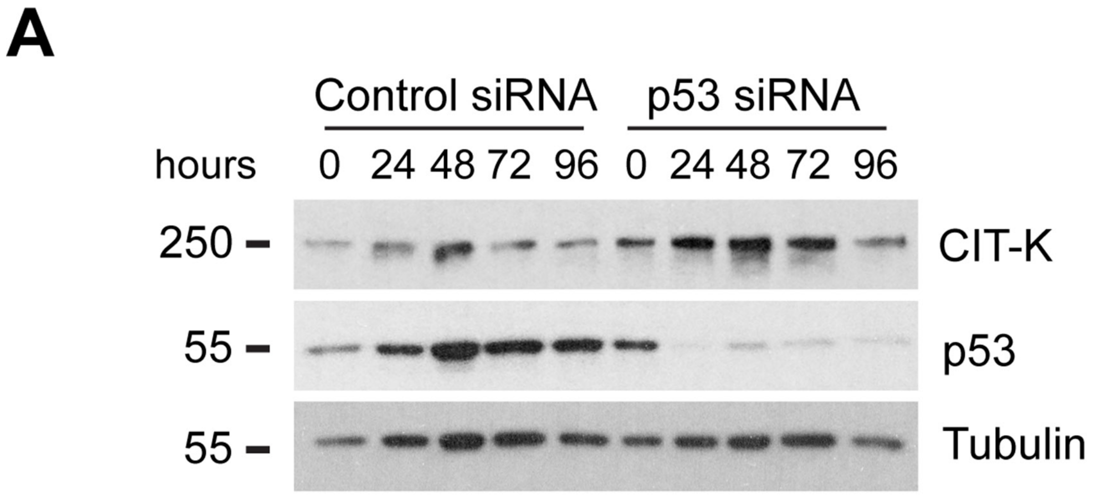


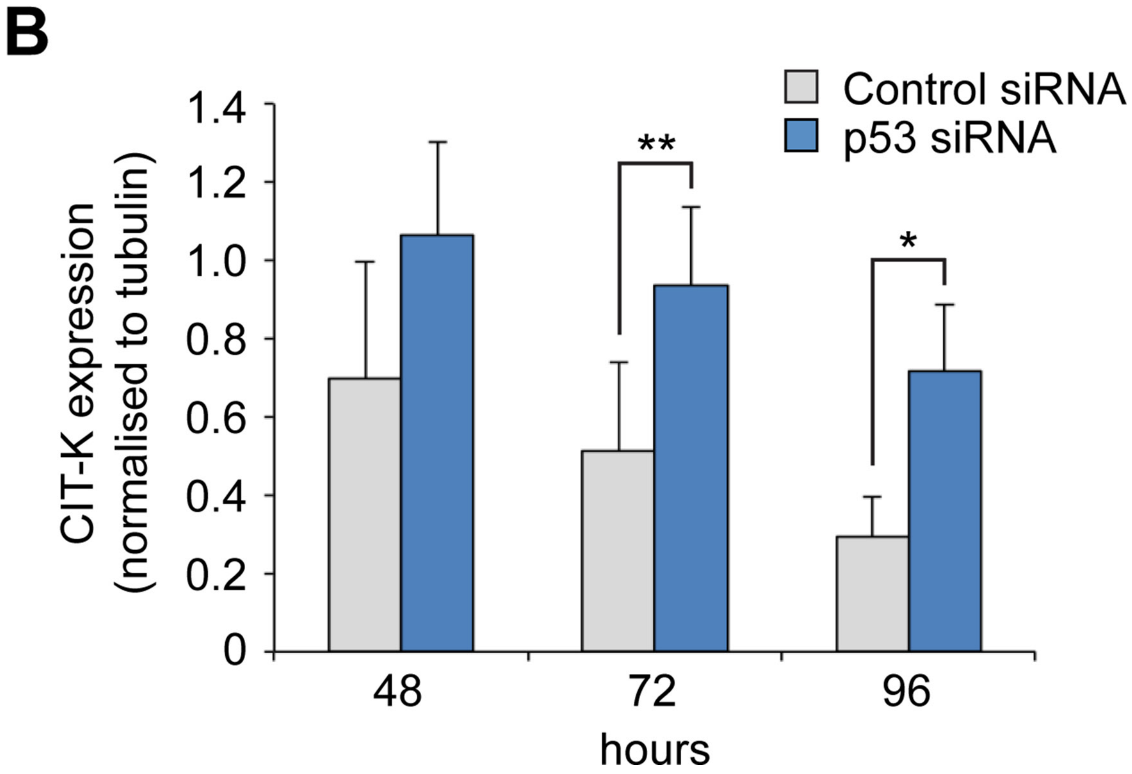


**Supplementary Figure S8: Knockdown of p53 increases expression of CIT-K. A.** HCT116 p53$^{+/+}$ cells were treated with either a control or p53 siRNA for up to 96 h, harvested and protein extracts analyzed by Western blot to detect CIT-K, p53 and tubulin (loading control). **B.** CIT-K expression from A was analysed by ImageJ and normalised to tubulin. Error bars represent SEM, n=7, * $p < 0.05$, ** $p < 0.01$ (Student's *t*-test).

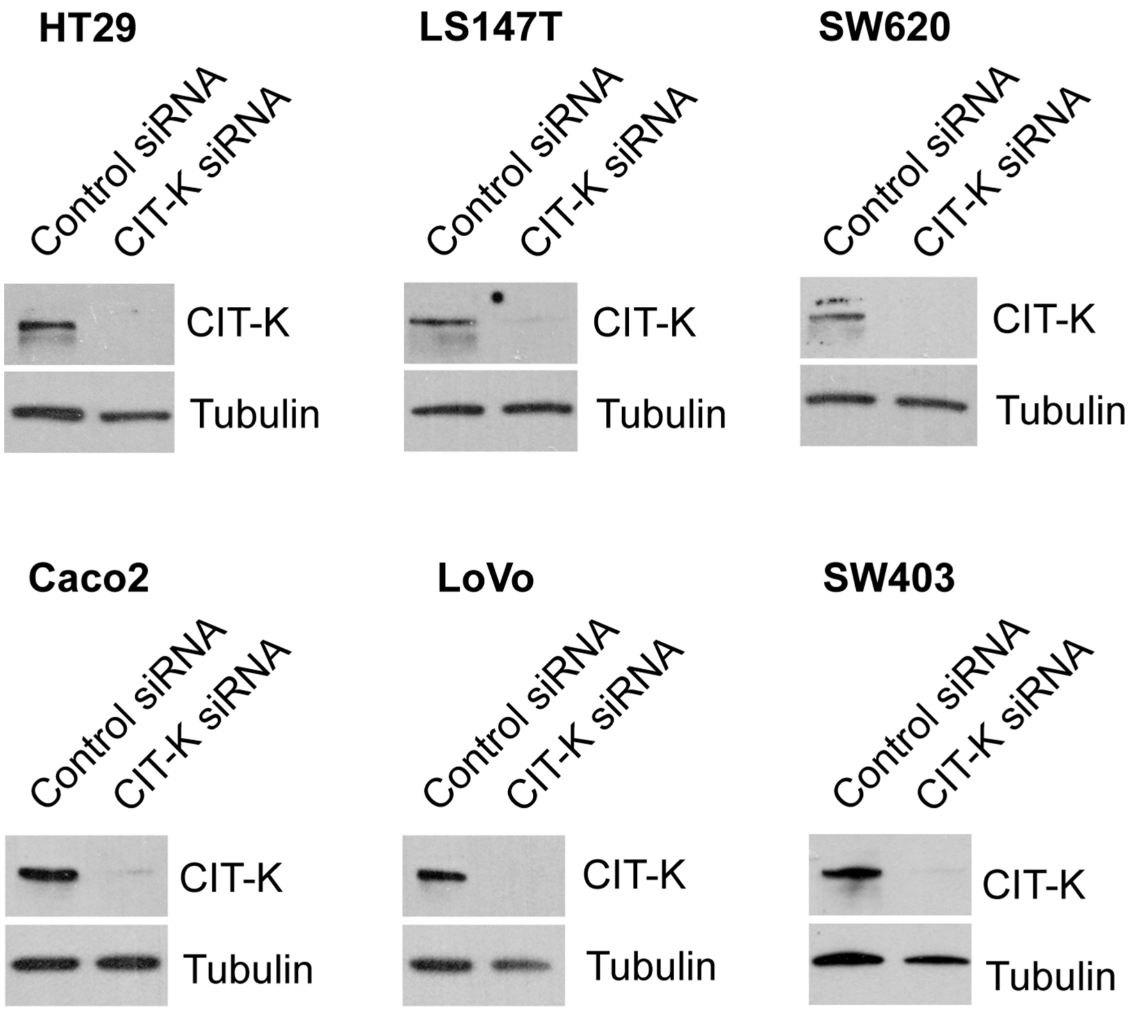


**Supplementary Figure S9: Knockdown of CIT-K via RNAi in HT29, LS147T, SW620, Caco2, LoVo and SW403 cells.** Cells were plated prior to transfection, treated with either control or CIT-K siRNA for 96 h, and then cells were harvested and protein extracts analyzed by Western blot to detect CIT-K and tubulin.
